# Ultrasound assessment of temporomandibular disorders: comparative analysis between inflammatory and degenerative patterns in rheumatic and non-rheumatic patients

**DOI:** 10.1007/s10067-025-07876-0

**Published:** 2025-12-18

**Authors:** Beatrice Maranini, Stefano Mandrioli, Giovanni Ciancio, Fabio Fabbian, Tommaso Bianchi, Manlio Galiè, Marcello Govoni

**Affiliations:** 1 Division of Rheumatology, Department of Medical Sciences, University of Ferrara, Ferrara, Italy; 2 Department of Cranio-Maxillofacial Surgery, Unit of Cranio-Maxillofacial Surgery, University of Ferrara, Ferrara, Italy; 3 Rheumatology Unit, Azienda Ospedaliero-Universitaria Policlinico di Modena, University of Modena and Reggio Emilia, Modena, Italy; 4 Department of Medical Sciences, University of Ferrara, Ferrara, Italy

**Keywords:** Inflammatory arthritis, Psoriatic arthritis, Rheumatoid arthritis, Temporomandibular joint, Ultrasound

## Abstract

**Background:**

Temporomandibular disorders (TMDs) encompass a range of conditions affecting the temporomandibular joint (TMJ), with causes that may be inflammatory or degenerative. Differentiating these mechanisms is essential for targeted treatment, especially in patients with underlying rheumatic diseases.

**Objective:**

This prospective, cross-sectional study assessed the utility of ultrasound (US) in distinguishing inflammatory from degenerative TMJ changes across different patient populations.

**Methods:**

From 2018 to February 2025, 272 patients with TMD symptoms underwent standardized TMJ US. They were grouped into those with inflammatory rheumatic diseases (*n* = 202), primary fibromyalgia (*n* = 33), and individuals without rheumatologic conditions (*n* = 37). A control group of 40 healthy subjects was also included. US findings assessed included joint effusion, synovial hypertrophy, power Doppler (PD) signal, erosions, condylar irregularities, and disc dislocation. Clinical symptoms and signs were also recorded.

**Results:**

Inflammatory findings (synovitis, effusion, PD signal) were significantly more frequent in the rheumatic group (*p* < 0.05). Physical exam alone could not reliably distinguish inflammatory from mechanical causes (66.4% vs 33.6%, *p* = 0.634). Degenerative changes were common in all groups, possibly reflecting chronic mechanical stress. PD signal appeared in some fibromyalgia cases, suggesting possible secondary inflammation. Articular erosions were the only US feature significantly correlated with symptoms.

**Conclusion:**

Ultrasound is a sensitive, non-invasive tool for assessing TMJ involvement, particularly in distinguishing inflammatory from mechanical patterns. Its integration into clinical evaluation improves diagnostic accuracy and informs more personalized management.

**Table:** 

**Key Points** • *Ultrasound (US) effectively distinguishes inflammatory from mechanical temporomandibular joint (TMJ) involvement, particularly in patients with autoimmune inflammatory rheumatic diseases (AIIRDs), outperforming clinical examination alone.* • *Inflammatory US features (e.g., joint effusion, synovial hypertrophy, power Doppler signal) were significantly more common in AIIRD patients, while degenerative features were present across all groups, including fibromyalgia and non-rheumatologic TMD.* • *Erosions were the only US finding significantly associated with TMJ symptoms, underscoring their potential clinical relevance in patient evaluation and management.* • *Integration of TMJ US into routine assessment improves diagnostic accuracy, supporting more personalized treatment strategies by identifying inflammatory versus mechanical patterns of TMJ involvement.*

**Supplementary Information:**

The online version contains supplementary material available at 10.1007/s10067-025-07876-0.

## Introduction

Temporomandibular disorders (TMDs) comprise heterogeneous conditions affecting the temporomandibular joint (TMJ), masticatory muscles, and related structures [1]. They represent a leading cause of non-dental orofacial pain worldwide, with a global prevalence estimated between 5 and 12% [2]. Typical symptoms—joint pain, limited mouth opening, joint noises, and muscular tenderness—impair function and quality of life [3].

TMJ involvement is increasingly recognized as a relevant manifestation in autoimmune inflammatory rheumatic diseases (AIIRDs) such as rheumatoid arthritis (RA) [4], juvenile idiopathic arthritis (JIA) [5], psoriatic arthritis (PsA) [6], ankylosing spondylitis (AS) [7], and connective tissue diseases (CTDs) [8, 9]. In these conditions, TMJ arthritis may be clinically silent but detectable by imaging, which can reveal early joint changes, highlighting the subclinical progression typical of early disease [10, 11]. If left untreated, TMJ involvement can lead to chronic pain, joint deformity, or ankylosis, particularly in younger patients [12].

Similar symptoms, however, also occur in non-rheumatologic TMD and in fibromyalgia syndrome (FMS), making clinical differentiation challenging [13, 14]. Overlapping clinical features blur diagnostic boundaries, as physical examination is often nonspecific and insufficient for determining etiology or disease severity [15, 16].

Magnetic resonance imaging (MRI) remains the gold standard for detecting TMJ soft tissue alterations, effusion, and synovial inflammation, but routine use is limited by cost, availability, and contraindications [17].

Musculoskeletal ultrasound (US) offers a non-invasive, dynamic, and widely available alternative for superficial structures such as synovium, joint capsule, cortical bone, and fluid collections [18, 19]. Power Doppler (PD) further enhances its diagnostic utility by revealing active synovial vascularization, a key marker of inflammation [20].

Despite growing interest, the use of TMJ US remains insufficiently standardized: challenges include anatomical complexity, operator dependency, and heterogeneity in scanning protocols [19]. Nevertheless, recent studies consistently show that US can reliably detect inflammatory TMJ changes and help distinguish them from degenerative alterations [21]. Yet few studies have systematically compared inflammatory versus mechanical patterns across rheumatic, fibromyalgic, and non-rheumatic TMD populations.

This study aimed to determine whether TMJ US can distinguish inflammatory from mechanical/degenerative alterations by comparing US findings across AIIRD, non-rheumatologic TMD, FMS, and healthy controls (HCs).

Secondary objectives were to evaluate possible correlations between US and clinical symptoms and to identify imaging patterns that may inform personalized management strategies.

## Materials and methods

This prospective, cross-sectional study was designed to evaluate the utility of US in distinguishing inflammatory from degenerative TMJ alterations in a clinical setting.

This study included patients consecutively evaluated between January 2018 and February 2025 at the dedicated Rheumatology–Maxillofacial joint outpatient Clinic for TMJ disorders at the University Hospital of Ferrara, reflecting a population with at least one TMD-related symptom.

Clinical examination of the TMJ was conducted by a maxillofacial surgeon followed by an US assessment of the affected region to confirm or exclude the presence of synovitis or other abnormalities (i.e., osteophytes, calcifications, or disc degeneration). All clinical and ultrasonographic examinations were performed by appropriately trained personnel with specific expertise in the assessment of temporomandibular disorders and musculoskeletal ultrasound, ensuring consistency and reliability of data acquisition.

In addition to this standard examination, all patients underwent a structured clinical assessment specifically focused on:

(1) TMJ pain, functional limitation, and joint sounds at physical examination.

(2) Bruxism.

(3) Evaluation of joint hyperlaxity.

(4) Number of tender and swelling joints.

(5) Clinimetric indexes (mean value, low/moderate/high disease activity).

The clinical picture of the studied cohort is shown in Supplementary Fig. S1.

TMJ US was performed by two blinded, experienced sonographers to reduce expectation bias; the Cohen’s kappa method was used to assess the degree of agreement among the two examiners (Supplementary Fig. S2).

The study design is shown in Supplementary Fig. S3.

### Inclusion criteria

- Patients meeting classification criteria for at least one AIIRD (e.g., PsA, AS, RA, JIA, Still’s disease, systemic lupus erythematosus (SLE), Sjögren’s syndrome, systemic sclerosis, dermatomyositis, undifferentiated CTD, granulomatosis with polyangiitis, or Behçet’s disease) and for FMS, based on established guidelines. All patients had at least one TMD-related symptom as the basis for their referral. Only adult participants with JIA diagnosis were included in the study to ensure a homogeneous sample and to avoid the anatomical and physiological variability of the temporomandibular joint observed during growth.
- Patients presenting with TMJ symptoms but without a diagnosed rheumatologic disease

All participants had to be over 18 years old and provide informed consent for clinical and US examination of the TMJ. Patients with JIA were included if they were over 18 years of age.

### Exclusion criteria

Patients were excluded if they had a history of significant dental alterations (e.g., prostheses, orthodontic appliances, malocclusion), prior TMJ surgeries for TMD, or non-rheumatologic TMJ conditions (e.g., severe craniofacial anomalies, traumatic TMD).

### Control group recruitment

Forty healthy subjects were recruited from the general population who did not have a history of inflammatory and/or degenerative rheumatic diseases and showed no signs or symptoms of TMJ involvement either at the time of enrollment or in their personal medical history.

### Data collection

The study protocol included three levels of evaluation:

1. Subjective evaluation:
  VAS pain during mastication (Visual Analog Scale from 0 to 10)
  Functional limitation (yes/no)
  Headache (yes/no)
2. Objective clinical evaluation by maxillofacial surgeon
  Clinical assessment of TMJ involvement aimed at detecting spontaneous or provoked pain on TMJ palpation, quantified using the VAS (0–10)
  Measurement of maximal spontaneous and forced mouth opening to assess functional limitation
  Evaluation of joint sounds (pops and clicks)
  Evaluation of pain at palpation of the temporal and/or masseter muscles
3. US evaluation

The US exam was performed with the patient in a supine position. The probe was placed parallel to the mandibular condyle and perpendicular to the zygomatic arch, using a linear transducer (12–18 MHz; Toshiba Aplio XG) (see Supplementary Fig. S4). For each TMJ (right and left), the following parameters were evaluated:

- Inflammatory findings: joint effusion, synovial hypertrophy, power Doppler (PD) signal.
  - We also evaluated articular cartilage for thinning and heterogeneous echotexture. PD signal was scored using the EULAR OMERACT scoring system (grade 0–3) [22]:
    - Grade 0: normal, absence of intra-articular flow signals
    - Grade 1: up to three single Doppler spots or up to one confluent spot and two single spots or up to two confluent spots
    - Grade 2: greater than grade 1 but < 50% Doppler signals in the total grey-scale background
    - Grade 3: greater than grade 2 (> 50% of the background grey-scale)
- Degenerative findings: condylar surface irregularities, enthesophytes, erosions, joint space narrowing, calcifications, disc dislocations, and reduced articular cartilage thickness (defined as qualitative assessment of thinning, heterogeneous echotexture).
- We also evaluated articular cartilage for thinning and heterogeneous echotexture. Cartilage thinning was defined as a reduction of the condyle–capsule distance below 1.4 mm, based on published reference data and confirmed by our control group findings [23]. Although validated cut-off values exist for hyaline cartilage thickness in other joints (e.g., metacarpal heads in rheumatoid arthritis), no validated or standardized cut-off for normal TMJ cartilage thickness has yet been established [24].

The terms “condylar surface irregularities” and “enthesophytes” and “osteophytes” were used interchangeably in some instances to describe osteoarthritic changes.

As a control group, 40 HCs without known inflammatory and/or degenerative rheumatic diseases and without signs and/or symptoms of TMJ involvement either at the time of enrollment or in their personal medical history were recruited.

In accordance with the Declaration of Helsinki, all patients gave informed consent before inclusion in the cohort. This study was approved by the local Institutional Ethics Review Board.

### Statistical analysis

The statistical analysis of this study was carried out using descriptive statistical methods. Categorical variables were expressed as frequencies and percentages, while all continuous variables with a Gaussian distribution were presented as mean ± standard deviation (SD).

A univariate analysis was performed for comparisons between the groups. The Student’s *t*-test for unpaired data was used to compare means for normally distributed variables; dichotomous variables were compared using the chi-square test.

Multiple regression models (multivariate analysis) were tested to examine the association between TMJ characteristics and both subjective and objective signs of involvement.

Odds ratios (ORs) and 95% confidence intervals were calculated to assess the independent association between the presence of specific US characteristics of the TMJ and the subjective and objective signs of involvement.

A *p*-value of < 0.05 was considered statistically significant.

## Results

### Descriptive analysis of the study population

The study involved 312 participants (see Table 1 for global population characteristics), including 202 patients with AIIRD (64.7%), 33 patients with primary FMS (10.6%), 37 patients without rheumatologic diseases presenting TMJ symptoms (11.9%), and 40 HCs (12.8%). Among patients with AIIRD, the most common condition was PsA (75.2%) (Supplementary Table S2; see also Supplementary Table S3 for HC group features).

**Table 1:** Descriptive table of the population under study (*n* = 312)

Parameter	Number (%)
AIIRD	202 (64.7%)
Primary FMS	33 (10.6%)
Non-rheumatological TMJ symptoms	37 (11.9%)
HCs	40 (12.8%)
Mean age of the entire population (years) ± standard deviation	56.2 ± 13.5
Mean age of the population with rheumatologic diseases (*n* = 202)	56.7 ± 12.1
Mean age of the population without rheumatologic diseases (*n* = 110)	56.2 ± 13.5
Gender (females/males)	215 (68.9%)/97 (31.1%)
Subjective symptoms related to the TMJ	162 (51.9%)
Objective signs related to the TMJ	137 (43.9%)
Joint effusion	142 (45.5%)
Synovial hyperplasia	58 (18.6%)
Erosions	40 (12.8%)
Power Doppler signal alterations	49 (15.7%)
Reduced articular cartilage thickness	64 (20.5%)
Articular cartilage echostructural heterogeneity	19 (6.1%)
Cortical bone irregularities	50 (16%)
Enthesophytes	64 (20.5%)
Calcifications	28 (8.9%)
Disc dislocations on the right side	9 (2.9%)
Disc dislocations on the left side	8 (2.6%)

It is important to note that, unlike other US findings which were analyzed as present or absent in either TMJ, disc dislocation was differentiated and recorded for the right and left sides. This distinction is clinically relevant, as disc dislocation is a highly specific marker of mechanical derangement. Reporting its laterality is crucial for informing targeted treatment, such as splint therapy or physical rehabilitation, which must be tailored to the affected side. This approach allows for a more precise assessment of the TMJ’s biomechanical function, which cannot be captured by analyzing the presence of the finding alone

*AIIRD*, autoimmune inflammatory rheumatic diseases; *FMS*, fibromyalgia syndrome; *HC*, healthy control; *TMJ*, temporomandibular joint

The mean age of the entire population was 56.2 ± 13.5 years, with a majority of females (68.9%) (Table 1).

51.9% of the entire population reported subjective TMJ symptoms, and 43.9% showed objective signs of TMJ involvement (Table 1).

US revealed inflammatory alterations in 48.7% of cases: joint effusion was detected in 45.5%, synovial hyperplasia in 18.6%, and PD-US changes in 15.7% (see Table 1).

Despite the absence of subjective symptoms or objective signs of TMJ involvement, 20% of HC displayed some US abnormalities, such as cartilage thinning (Supplementary Table S3).

### Rheumatologic and non-rheumatologic patient comparisons

In AIIRD, most frequent US changes were joint effusion (56.4%), synovial hyperplasia (24.8%), and increased PD (22.8%), followed by erosions (16.8%) and other structural alterations such as enthesophytes (19.8%), reduced articular cartilage thickness (16.3%), cortical bone irregularities (13.9%), calcifications (10.4%), articular cartilage echostructural heterogeneity (8.4%), and right (1.5%) and left (1%) disc dislocations (Supplementary Table S4).

All patients with primary fibromyalgia were female, with 100% reporting subjective symptoms. 54.5% showed objective signs of TMJ involvement. Degenerative changes such as reduced cartilage thickness and cortical irregularities were more common than inflammatory signs (Supplementary Table S5).

97.3% of TMD patients without rheumatic conditions reported subjective symptoms, and 75.7% had objective TMJ signs. The most common US findings included joint effusion (43.2%) and reduced cartilage thickness (32.4%). A positive PD signal was found in only one case (a patient also exhibited joint effusion and marked cortical irregularities) (Supplementary Table S6).

### US findings comparison

**Fig. 1:**
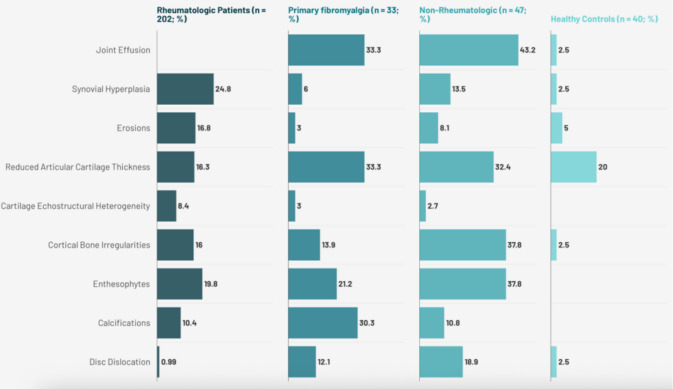
A line chart depicting the comparison of US findings among rheumatologic patients, fibromyalgia patients, non-rheumatologic patients, and healthy controls

Figure 1 compares US findings among patients with AIIRD, FMS, non-rheumatologic TMD, and HCs. AIIRD showed higher rates of joint effusion and erosions, whereas FMS patients frequently exhibited cartilage thinning. Non-rheumatologic TMD patients had common cartilage and bone alterations, including cortical irregularities and enthesophytes.

**Fig. 2:**
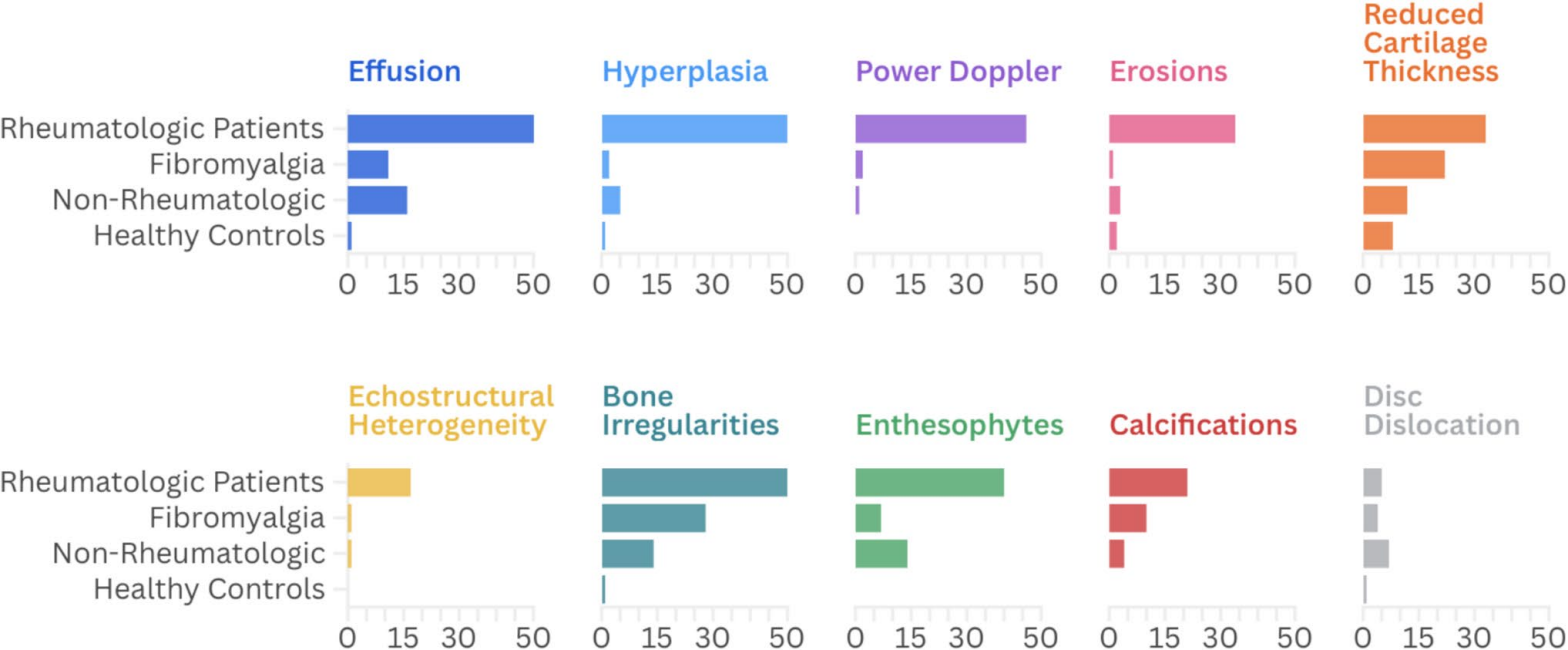
Grid of bar charts showing a visual distribution (in absolute values) of the US manifestations analyzed in the studied patients

Grid of bar charts in Fig. 2 visually represents the distribution of various US manifestations across patient groups.

### Comparative analysis between populations

#### Univariate analysis comparing clinical and US findings between primary FMS + non-rheumatologic TMD patients + HC (Group A) versus AIIRD patients (Group B) (Table 2)

**Table 2:** Univariate analysis between two populations: primary fibromyalgia, healthy controls, and patients with TMD but no rheumatologic pathology diagnosis (Group A) versus patients with chronic inflammatory diseases (arthritis, connective tissue diseases, or vasculitis) (Group B)

	Group A (*n* = 110)	Group B (*n* = 202)	*p*-value
Age (years)	56.2 ± 13.5	56.7 ± 12.1	0.732
Male/female	15/95 (15.5%/44.2%)	82/120 (84.5%/55.8%)	< 0.0001
Subjective TMJ symptoms	69 (42.6%)	93 (57.4%)	0.006
Objective TMJ signs	46 (33.6%)	91 (66.4%)	0.634
Joint effusion	28 (19.7%)	114 (80.3%)	< 0.001
Synovial hyperplasia	8 (13.8%)	50 (86.2%)	< 0.0001
Erosions	6 (15%)	34 (85%)	0.004
Power Doppler signal alterations	3 (6.1%)	46 (93.9%)	< 0.0001
Reduced articular cartilage thickness^a^	31 (48.4%)	33 (51.6%)	0.018
Articular cartilage echostructural heterogeneity	2 (10.5%)	17 (89.5%)	0.024
Cortical bone irregularities	22 (44%)	28 (56%)	0.196
Enthesophytes	24 (37.5%)	40 (62.5%)	0.663
Calcifications	7 (25%)	21 (75%)	0.301
Disc dislocations on the right side	6 (66.7%)	3 (33.3%)	0.072
Disc dislocations on the left side	6 (75%)	2 (25%)	0.025

*TMJ*, temporomandibular joint

^a^The percentage of reduced articular cartilage thickness was equally distributed in the two groups; however, this echographic alteration was higher in the groups without chronic inflammatory diseases (28.2% vs 16.3%, *p* = 0.018)

Group B had a slightly higher average age than Group A, though the difference was not statistically significant. A significant gender disparity was observed, with a higher proportion of females in both groups, especially in Group A (*p* < 0.0001).

A significantly higher percentage of patients in Group B reported subjective TMJ symptoms compared to Group A (*p* < 0.001). There is no significant difference in the presence of objective TMJ signs between the two groups.

A significantly higher percentage of Group B patients showed joint effusion (*p* < 0.001), synovial hyperplasia (*p* < 0.0001), PD alterations (*p* < 0.0001), and erosions (*p* = 0.004) in the TMJ compared to Group A.

Although the percentage of reduced articular cartilage thickness was equally distributed in the two groups, this echographic alteration was higher in the groups without chronic inflammatory diseases (28.2% vs 16.3%, *p* = 0.018). Cartilage echostructural heterogeneity was more frequent in Group B (*p* = 0.024); moreover, Group B had more frequent enthesophytes, though the difference was not statistically significant.

No significant differences in terms of cortical bone irregularities and calcifications were observed between the groups.

Left-sided disc dislocations were significantly more common in Group A (*p* = 0.025), while no significant differences were found for right-sided dislocations.

In the multivariate analysis model, we included variables that exhibited a statistically significant difference in the univariate analysis between the two patient groups (Group A and Group B), namely, joint effusion, synovial hyperplasia, presence of erosions, PD signal alterations, reduction in articular cartilage thickness, and echostructural heterogeneity.

Logistic regression analysis with “subjective symptoms of TMJ involvement” as the dependent variable revealed a significant association between subjective symptoms and two US findings: the presence of erosions (odds ratio (OR), 2.263; 95% confidence interval (CI 95%), 1.058–4.838;

*p*-value, 0.035) and a reduction in articular cartilage thickness (OR, 0.281; CI 95%, 0.148–0.536; *p*-value, < 0.0001) (Table 3).

**Table 3:** Association between subjective symptoms of TMJ involvement and US manifestations

Parameter	OR	95% CI	*p*-value
Erosions	2.263	1.058–4.838	0.035
Reduction in articular cartilage thickness	0.281	0.148–0.536	< 0.0001

*CI 95%*, 95% confidence interval; *OR*, odds ratio

The logistic regression analysis with the parameter “objective signs of TMJ involvement” shows an association between objective signs of TMJ involvement and the reduction in articular cartilage thickness (OR, 0.3; 95% CI, 0.164–0.549; *p*-value, < 0.001) (Table 4).

**Table 4:** Association between objective signs of TMJ involvement and US manifestations

Parameter	OR	95% CI	*p*-value
Reduction in articular cartilage thickness	0.3	0.164–0.549	< 0.0001

*CI 95%*, 95% confidence interval; *OR*, odds ratio

#### Univariate analysis comparing AIIRD versus primary FMS, non-rheumatologic TMD patients versus primary FMS, AIIRD versus non-rheumatologic TMD patients, non-rheumatologic TMD patients versus HCs, and primary FMS versus HCs is shown in Supplementary Tables S7–S11, respectively.

From Supplementary Table S7 onwards, we present a series of univariate comparative analyses between different patient populations. These analyses aim to compare and contrast key clinical and US findings among the following groups:

- Supplementary Table S7: univariate analysis comparing AIIRD versus primary FMS
- Supplementary Table S8: AIIRD versus non-rheumatologic TMD patients
- Supplementary Table S9: non-rheumatologic TMD patients versus primary FMS
- Supplementary Table S10: non-rheumatologic TMD patients versus HCs
- Supplementary Table S11: primary FMS versus HCs

## Discussion

MRI is still considered the gold standard in the study of inflammatory and degenerative alterations of the TMJ [18, 25–27], but US is increasingly recognized as a valuable, rapid, and accessible complementary tool [19, 28]. Especially during specialist consultations, US improves diagnostic accuracy when combined with physical examination, particularly in cases with nonspecific symptoms or suspected inflammatory arthropathies [29].

Importantly, we emphasize that ultrasound cannot replace a comprehensive musculoskeletal assessment. The clinical examination of the TMJ—encompassing symptoms, functional limitations, stiffness, and ligamentous hyperlaxity—is essential for accurate interpretation of US findings. Ultrasound should therefore be integrated into, rather than substituted for, clinical evaluation.

In this large, multidisciplinary cohort, we have found a higher frequency of subjective TMJ symptoms in patients with AIIRD. However, physical examination alone did not reliably discriminate inflammatory from mechanical involvement.

Nonetheless, clinical characteristics such as joint stiffness—typical of rheumatoid arthritis and often reflecting disease activity—and ligamentous hyperlaxity—common in both rheumatic and non-rheumatic patients and associated with early osteoarthritis—were systematically recorded and contextualized when interpreting US findings.

US proved more sensitive and specific in detecting signs of active inflammation—effusion, synovial hypertrophy, and PD signal—especially in the AIIRD group, thus contributing significantly to distinguishing inflammatory from mechanical involvement. These findings support the concept that US adds clinically relevant information beyond physical examination, particularly for identifying subclinical or early inflammatory involvement in AIIRD.

Degenerative/structural changes (e.g., cortical irregularities, enthesophytes) were also common in AIIRD patients. This can be explained by inflammation-driven degenerative pathways in a joint with thin fibrocartilage which can act independently of mechanical stress to promote degenerative changes in a joint with thin fibrocartilage. The TMJ, in fact, presents a unique anatomical model for studying osteoarthritis due to its thin fibrocartilage covering, which leaves the underlying bone more exposed to damage [30]. Therefore, the TMJ’s structure not only predisposes it to injury but also amplifies the impact of inflammatory processes that drive osteoarthritic damage, as seen in other synovial joints [31]. This supports the heterogeneity of the pathogenic mechanisms underlying osteoarthritic changes [32]. The course of TMJ pathology differs between rheumatic and non-rheumatic disorders: patients with ligamentous hypermobility or functional overload (e.g., bruxism) may develop osteoarthritic changes via mechanisms distinct from inflammation-driven pathways seen in AIIRD. The integration of clinical features in our assessment is therefore crucial for interpreting these heterogeneous degenerative profiles.

Cortical irregularities, enthesophytes, and meniscal calcifications did not show significant differences between patients with chronic inflammatory rheumatic disease and FMS alone. In the last group, the mechanisms underlying the degenerative changes are very frequent and not inflammatory, but primarily functional, as seen in bruxism [14, 33].

Among mechanical changes, only disc displacement was found to be more frequent in patients with non-rheumatic TMD compared to those with chronic inflammatory rheumatic disease. Unlike inflammation-driven changes, such as synovitis or bone erosion, disc displacement involves the structural positioning of the disc within the joint, without the inflammatory processes that cause joint swelling.

Interestingly, effusion and erosions were also observed in non-rheumatic TMD, suggesting these changes may result from mechanical joint stress. In fact, disc displacements, intra-articular calcifications, and heterogeneous cartilage echotexture can cause reactive capsular distension, which, if not recognized and treated, may progress to condylar erosions even in the absence of true joint inflammation [21]. These findings further support the necessity of combining imaging with clinical status, as purely mechanical dysfunctions (e.g., disc displacement or hypermobility-related instability) may lead to reactive capsular changes that mimic inflammatory features on US.

However, when comparing non-rheumatic TMD + FMS as a group, the distinction from AIIRD became less clear, likely due to the low inflammatory burden in FMS patients (see Table 2). This observation currently lacks a clear explanation but certainly warrants confirmation in studies with larger sample sizes, to explain even erosions’ findings.

In contrast, comparison with HC, representing the general population, highlights a significantly higher number of degenerative US findings (cortical irregularities, enthesophytes, calcifications, and disc displacements) in TMD patients.

Few FMS patients showed PD signals, likely reflecting reactive hyperemia from advanced degeneration, rather than true synovitis. Indeed, it is known that the degenerative-arthritic process can be complicated by secondary inflammation due to the debridement of arthritic fragments into the joint space [34].

AIIRD patients showed more cartilage heterogeneity, possibly indicating early damage [21].

Multivariate analysis revealed that only erosions correlated with symptoms, while other findings (e.g., effusion, PD signal, cartilage thinning) did not. Some US changes may thus represent early, subclinical damage or even normal variants. It is worth noting that cartilage thinning, although also observed in about 20% of healthy subjects, did not correlate with symptoms or signs in this context, confirming that some US findings may represent physiological variants without pathological significance.

Taken together, our findings highlight the complementary role of ultrasound in the assessment of TMJ involvement across different disease contexts. While MRI remains the gold standard for evaluating inflammatory and degenerative alterations of the TMJ, ultrasound offers a valuable, rapid, and accessible imaging modality that can be easily implemented in specialist consultations to enhance diagnostic accuracy.

In light of our results and current evidence, a stepwise ultrasonographic approach could be proposed, improving the standardization and clinical applicability of TMJ ultrasound. The first step should focus on identifying the diagnostic triad—effusion, synovial hypertrophy, and PD signal—as the core features suggestive of active synovitis. The second step should evaluate structural changes such as cartilage thinning, cortical irregularities, and enthesophytes, which may indicate chronic or degenerative remodeling. The third step involves assessment of disc position and mobility to identify mechanical dysfunctions such as displacement. This stepwise approach is designed to be explicitly combined with structured clinical examination, including evaluation of stiffness, hyperlaxity, and functional limitation, ensuring that US findings are interpreted within each patient’s clinical context. These imaging findings should then be integrated with clinical assessment to distinguish inflammatory from mechanical or functional involvement.

This structured framework may facilitate reproducibility among operators with different levels of experience and promote broader use of ultrasound in the diagnostic work-up of TMJ disorders. Furthermore, it underscores the potential of ultrasound as a practical bridge between clinical evaluation and MRI, particularly useful for early detection and longitudinal monitoring of inflammatory TMJ involvement in AIIRD and other systemic conditions.

Methodologically, strengths include the multidisciplinary setting and large sample size. Limitations include the cross-sectional design, potential operator dependence, lack of MRI in all participants (precluding definitive sensitivity/specificity estimates), and possible group imbalance (e.g., high PsA proportion). Future studies should adopt standardized TMJ US protocols (including PD parameters) and validate longitudinally whether US phenotypes predict outcomes and treatment response.

## Conclusions

TMJ US, particularly the combination of synovitis/effusion/PD, improves the discrimination of inflammatory versus mechanical TMJ involvement beyond clinical examination. Erosions carry symptomatic relevance, whereas cartilage thinning is common and nonspecific. Integrating TMJ US into routine assessment may help separate inflammatory from mechanical phenotypes when symptoms are nonspecific, prioritize rheumatologic management when PD/synovitis/effusion is present, prompt dental/physiotherapy pathways for predominantly mechanical patterns (e.g., disc displacement, enthesophytes, cortical irregularities), and identify patients with erosions who are more likely to be symptomatic and may benefit from closer monitoring. This integrated approach may guide referrals, prioritize rheumatologic treatment when inflammatory markers are detected, and prevent unnecessary imaging when a mechanical pattern predominates.

Further prospective studies with larger and long-term cohorts will be essential to validate these findings, deepen the understanding of the pathophysiological mechanisms underlying TMJ US alterations, and more precisely define the role of US in risk stratification and personalized treatment planning.

## Supplementary Information

Below is the link to the electronic supplementary material.

ESM 1 (DOCX 909 KB)

## Data Availability

The original contributions presented in the study are included in the article/supplementary material. Further inquiries can be directed to the corresponding author.
