# Supplementary material for: Ultrasound assessment of temporomandibular disorders: comparative analysis between inflammatory and degenerative patterns in rheumatic and non-rheumatic patients
Source: Clin Rheumatol. 2025 Dec 18;45(3):1993–2002. doi: 10.1007/s10067-025-07876-0 (PMC12923428; doi:10.1007/s10067-025-07876-0)
Supplement: Supplementary file 1 — (DOCX 909 KB) [file 10067_2025_7876_MOESM1_ESM.docx]

Supplementary Material

**Supplementary Fig. S1.** Inter-rater reliability (Cohen's Kappa) for ultrasound findings.

This table presents the Cohen's Kappa (κ) values for the inter-rater reliability of key ultrasound manifestations. The kappa values quantify the agreement between two independent observers beyond what would be expected by chance. A Kappa score greater than 0.80 indicates almost perfect agreement, while a score between 0.61 and 0.80 signifies substantial agreement. Findings such as Joint Effusion and Positive Power Doppler showed high to almost perfect agreement, confirming their objective nature and the consistency of the raters' assessments. Conversely, Reduced Cartilage Thickness showed moderate agreement, indicating that this is a more subjective finding.

| **US Manifestation** | **Kappa (κ) Value** | **Interpretation** |
| --- | --- | --- |
| **Inflammatory Findings** | | |
| Joint Effusion | 1.24 | Almost perfect agreement |
| Synovial Hypertrophy | 1.18 | Substantial agreement |
| Positive Power Doppler | 1.21 | Almost perfect agreement |
| **Degenerative Findings** | | |
| Cortical Irregularities | 1.25 | Almost perfect agreement |
| Osteophytes | 1.18 | Substantial agreement |
| Reduced Cartilage Thickness | 0.59 | Moderate agreement |
| Disc Dislocation | 1.31 | Almost perfect agreement |

**Supplementary Fig. S2.** Study design description. All subjects recruited were patients referred to the Rheumatology–Maxillofacial Clinic for TMJ disorders at the University Hospital of Ferrara.

Clinical examination and US were performed separately.

All data were collected in accordance with protocols reported in previous studies (1-5).

TMJ: temporomandibular joint; US: ultrasound.


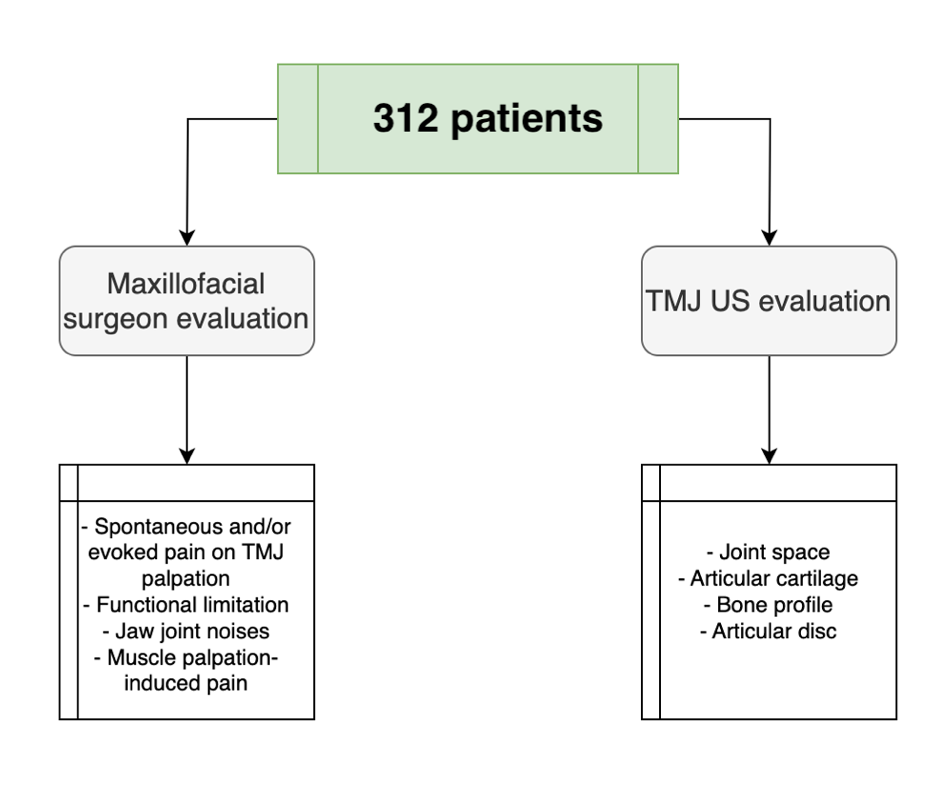


**Supplementary Fig. S3.** TMJ US technique. Conventional TMJ US longitudinal scan, with transducer parallel to the ramus of mandible, in closed-mouth position. Personal archive.

TMJ: temporomandibular joint; US: ultrasound.


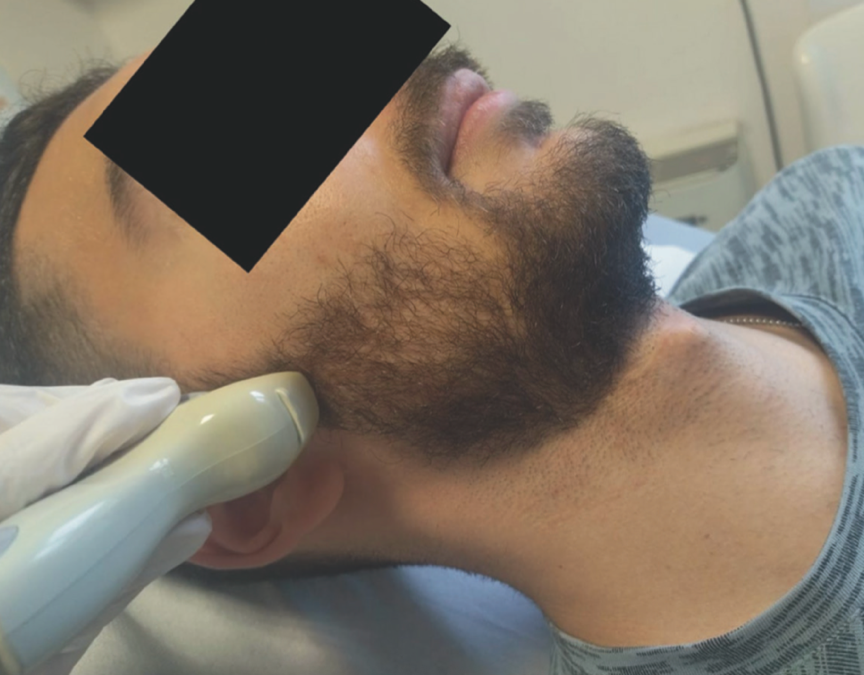


**Supplementary Table S1**. Summary of clinical findings collected during TMJ assessment: TMJ pain, functional limitation, and joint sounds; presence of bruxism; evaluation of joint hyperlaxity; tender and swollen joint counts; and clinimetric indexes of disease activity (mean value and distribution across low, moderate, and high activity).

SJC: Swollen Joint Count; TMD: Temporomandibular Disorder; TJC: Tender Joint Count; TMJ: Temporomandibular Joint.

|  | **TMD without known rheumatologic pathology** | **Primary fibromyalgia** | **Chronic inflammatory disease (arthritis, connective tissue disease, or vasculitis)** | **Healthy control population** |
| --- | --- | --- | --- | --- |
| **TMJ pain** | 23 (62.2%) | 17 (51.5%) | 164 (81.2%) | 0 (0%) |
| **TMJ functional limitation** | 16 (43.2%) | 8 (24.2%) | 36 (17.8%) | 0 (0%) |
| **TMJ sounds** | 12 (32.4%) | 8 (24.2%) | 44 (21.8%) | 0 (0%) |
| **Bruxism** | 12 (32.4%) | 15 (45.4%) | 82 (40.6%) | 0 (0%) |
| **Joint hyperlaxity** | 1 (2.7%) | 0 (0%) | 0 (0%) | 0 (0%) |
| **Mean TJC** | 0 | 0 | 1.08 | 0 |
| **Mean SJC** | 0 | 0 | 0.5 | 0 |
| **CLINIMETRIC INDEXES IN RHEUMATOLOGICAL INFLAMMATORY CONDITIONS** | | | | |
|  | **Remission** | **Low disease activity** | **Moderate disease activity** | **High disease activity** |
| **Joint effusion and/or synovial hyperplasia (n=121)** | 46 (38%) | 50 (41.3%) | 21 (17.4%) | 4 (3.3%) |
| **No joint effusion or synovial hyperplasia (n=81)** | 52 (64.2%) | 18 (22.2%) | 10 (12.3%) | 1 (1.2%) |

**Supplementary Table S2.** Chronic inflammatory rheumatic population under study and percentage of each out of the total.

AS: Ankylosing Spondylitis; DM: Dermatomyositis; GPA: Granulomatosis with Polyangiitis; JIA: Juvenile Idiopathic Arthritis; PsA: Psoriatic Arthritis; RA: Rheumatoid Arthritis; SjS: Sjögren's syndrome; SLE: Systemic Lupus Erythematosus; SSc: Systemic Sclerosis; UCTD: Undifferentiated Connective Tissue Disease.

| **Rheumatologic Condition** | **Number of Cases** | **%** |
| --- | --- | --- |
| Psoriatic arthritis (PsA) | 152 | 75.2% |
| Rheumatoid arthritis (RA) | 23 | 11.4% |
| Systemic lupus erythematosus (SLE) | 5 | 2.5% |
| Ankylosing spondylitis (AS) | 2 | 0.9% |
| Sjögren's syndrome (SjS) | 6 | 2.9% |
| Undifferentiated connective tissue disease (UCTD) | 4 | 1.9% |
| Juvenile idiopathic arthritis (JIA) | 4 | 1.9% |
| Dermatomyositis (DM) | 1 | 0.5% |
| Granulomatosis with polyangiitis (GPA) | 1 | 0.5% |
| Systemic sclerosis (SSc) | 2 | 0.9% |
| Behçet’s disease | 1 | 0.5% |
| Still’s disease | 1 | 0.5% |
| Rheumatologic disease with concomitant secondary fibromyalgia | 13 | 6.4% |

**Supplementary Table S3.** Characteristics of healthy control population.

TMJ: Temporomandibular Joint.

| **Parameter** | **Number of Cases** |
| --- | --- |
| Mean age ± standard deviation | 44 ± 1.4 |
| Sex (females/males) | 28 (70%) / 12 (30%) |
| Subjective symptoms related to TMJ | 0 (0%) |
| Objective signs related to TMJ | 0 (0%) |
| Joint effusion | 1 (2.5%) |
| Synovial hyperplasia | 1 (2.5%) |
| Erosions | 2 (5%) |
| Power Doppler signal alterations | 0 (0%) |
| Reduction in articular cartilage thickness | 8 (20%) |
| Echostructural heterogeneity in articular cartilage | 0 (0%) |
| Cortical bone irregularities | 1 (2.5%) |
| Enthesophytes | 0 (0%) |
| Calcifications | 0 (0%) |
| Disc dislocations on the right | 0 (0%) |
| Disc dislocations on the left | 1 (2.5%) |

**Supplementary Table S4.** Patients with chronic inflammatory disease (arthritis, connective tissue disease, or vasculitis).

TMJ: Temporomandibular Joint.

| **Parameter** | **Number of Cases** |
| --- | --- |
| Mean age (years) ± standard deviation | 56.7 ± 12.1 |
| Gender (female/male) | 120 (59.4%) / 82 (40.6%) |
| Subjective symptoms related to the TMJ | 93 (46%) |
| Objective signs related to the TMJ | 91 (45%) |
| Joint effusion | 114 (56.4%) |
| Synovial hyperplasia | 50 (24.8%) |
| Erosions | 34 (16.8%) |
| Power Doppler signal alterations | 46 (22.8%) |
| Reduction in articular cartilage thickness | 33 (16.3%) |
| Echostructural heterogeneity in articular cartilage | 17 (8.4%) |
| Cortical bone irregularities | 28 (13.9%) |
| Enthesophytes | 40 (19.8%) |
| Calcifications | 21 (10.4%) |
| Disc dislocations on the right | 3 (1.5%) |
| Disc dislocations on the left | 2 (1%) |

**Supplementary Table S5.** Patients with primary fibromyalgia.

TMJ: Temporomandibular Joint.

| **Parameter** | **Number of Cases** |
| --- | --- |
| Mean age (years) ± standard deviation | 55.3 ± 40.4 |
| Gender (female/male) | 33 (100%) / 0 |
| Subjective symptoms related to the TMJ | 33 (100%) |
| Objective signs related to the TMJ | 18 (54.5%) |
| Joint effusion | 11 (33.3%) |
| Synovial hyperplasia | 2 (6%) |
| Erosions | 1 (3%) |
| Power Doppler signal alterations | 2 (6%) |
| Reduction in articular cartilage thickness | 11 (33.3%) |
| Echostructural heterogeneity in articular cartilage | 1 (3%) |
| Cortical bone irregularities | 7 (21.2%) |
| Enthesophytes | 10 (30.3%) |
| Calcifications | 3 (9%) |
| Disc dislocations on the right | 2 (6%) |
| Disc dislocations on the left | 2 (6%) |

**Supplementary Table S6.** Patients with TMD without known rheumatologic pathology.

TMJ: Temporomandibular Joint.

| **Parameter** | **Number of Cases** |
| --- | --- |
| Average age (years) ± standard deviation | 71.6 ± 15.8 |
| Sex (female/male) | 34 (91.9%) / 3 (8.1%) |
| Subjective symptoms related to the TMJ | 36 (97.3%) |
| Objective signs related to the TMJ | 28 (75.7%) |
| Joint effusion | 16 (43.2%) |
| Synovial hyperplasia | 5 (13.5%) |
| Erosions | 3 (8.1%) |
| Power Doppler signal alterations | 1 (2.7%) |
| Reduction in articular cartilage thickness | 12 (32.4%) |
| Echostructural heterogeneity in articular cartilage | 1 (2.7%) |
| Cortical bone irregularities | 14 (37.8%) |
| Enthesophytes | 14 (37.8%) |
| Calcifications | 4 (10.8%) |
| Disc dislocations on the right | 4 (10.8%) |
| Disc dislocations on the left | 3 (8.1%) |

**Supplementary Table S7.** Univariate comparative analysis between autoimmune inflammatory rheumatic diseases (AIIRD) *versus* primary Fibromyalgia syndrome (FMS).

TMJ: Temporomandibular Joint.

|  | **Fibromyalgia (n = 33)** | **Rheumatologic Patients with Arthritic/Connective Tissue/Vasculitis Disease (n = 202)** | **p value** |
| --- | --- | --- | --- |
| Age (years) | 59.7 ± 14.2 | 56.7 ± 12.1 | 0.199 |
| Male/Female | 0/33 (0%/21.6%) | 82/120 (100%/78.4%) | <0.0001 |
| Subjective TMJ symptoms | 33 (26.2%) | 93 (73.8%) | <0.001 |
| Objective TMJ signs | 18 (16.5%) | 91 (83.5%) | 0.350 |
| Joint effusion | 11 (8.8%) | 114 (91.2%) | 0.015 |
| Synovial hyperplasia | 2 (3.8%) | 50 (96.2%) | 0.013 |
| Erosions | 1 (2.9%) | 34 (97.1%) | 0.036 |
| Power Doppler signal alterations | 2 (4.2%) | 46 (95.8%) | 0.034 |
| Reduction in articular cartilage thickness | 11 (25%) | 33 (75%) | 0.029 |
| Echostructural heterogeneity in articular cartilage | 1 (5.6%) | 17 (94.4%) | 0.481 |
| Cortical bone irregularities | 7 (20%) | 28 (80%) | 0.292 |
| Enthesophytes | 10 (20%) | 40 (80%) | 0.175 |
| Calcifications | 3 (12.5%) | 21 (87.5%) | 1.000 |
| Disc dislocations on the right | 2 (40%) | 3 (60%) | 0.146 |
| Disc dislocations on the left | 2 (50%) | 2 (50%) | 0.096 |

**Supplementary Table S8.** Univariate comparative analysis between AIIRD *versus* non-rheumatologic TMD patients.

TMJ: Temporomandibular Joint.

|  | **Patients without Rheumatologic Pathology (n = 37)** | **Rheumatologic Patients with Arthritic/Connective Tissue/Vasculitis Disease (n = 202)** | **p value** |
| --- | --- | --- | --- |
| Age (years) | 58.3 ± 13.7 | 56.7 ± 12.1 | 0.480 |
| Male/Female | 3/34 (3.5%/22.1%) | 82/120 (96.5%/77.9%) | <0.0001 |
| Subjective TMJ symptoms | 36 (27.9%) | 93 (72.1%) | <0.001 |
| Objective TMJ signs | 28 (23.5%) | 91 (76.5%) | 0.001 |
| Joint effusion | 16 (12.3%) | 114 (87.7%) | 0.154 |
| Synovial hyperplasia | 5 (9.1%) | 50 (90.9%) | 0.201 |
| Erosions | 3 (8.1%) | 34 (91.9%) | 0.222 |
| Power Doppler signal alterations | 1 (2.1%) | 46 (97.9%) | 0.003 |
| Reduction in articular cartilage thickness | 12 (26.7%) | 33 (73.3%) | 0.037 |
| Echostructural heterogeneity in articular cartilage | 1 (5.6%) | 17 (94.4%) | 0.322 |
| Cortical bone irregularities | 14 (33.3%) | 28 (66.7%) | 0.001 |
| Enthesophytes | 14 (25.9%) | 40 (74.1%) | 0.030 |
| Calcifications | 4 (16%) | 21 (84%) | 1.000 |
| Disc dislocations on the right | 4 (57.1%) | 3 (42.9%) | 0.012 |
| Disc dislocations on the left | 3 (60%) | 2 (40%) | 0.028 |

**Supplementary table S9.** Univariate comparative analysis between non-rheumatologic TMD patients *versus* primary FMS.

TMJ: Temporomandibular Joint.

|  | **Patients without Rheumatologic Pathology (n = 37)** | **Fibromyalgia (n = 33)** | **p value** |
| --- | --- | --- | --- |
| Age (years) | 58.3 ± 13.7 | 59.7 ± 14.2 | 0.667 |
| Male/Female | 3/34 (100%/50.7%) | 0/33 (0%/49.3%) | 0.242 |
| Subjective TMJ symptoms | 36 (52.2%) | 33 (47.8%) | 1.000 |
| Objective TMJ signs | 28 (60.9%) | 18 (39.1%) | 0.080 |
| Joint effusion | 16 (59.3%) | 11 (40.7%) | 0.465 |
| Synovial hyperplasia | 5 (71.4%) | 2 (28.6%) | 0.434 |
| Erosions | 3 (75%) | 1 (25%) | 0.616 |
| Power Doppler signal alterations | 1 (33.3%) | 2 (66.7%) | 0.599 |
| Reduction in articular cartilage thickness | 12 (52.2%) | 11 (47.8%) | 1.000 |
| Echostructural heterogeneity in articular cartilage | 1 (50%) | 1(50%) | 1.000 |
| Cortical bone irregularities | 14 (66.7%) | 7 (33.3%) | 0.192 |
| Enthesophytes | 14 (58.3%) | 10 (41.7%) | 0.616 |
| Calcifications | 4 (57.1%) | 3 (42.9%) | 1.000 |
| Disc dislocations on the right | 4(66.7%) | 2 (33.3%) | 0.677 |
| Disc dislocations on the left | 3 (60%) | 2 (40%) | 1.000 |

**Supplementary table S10.** Univariate comparative analysis between non-rheumatologic TMD patients *versus* healthy control group (HC).

TMJ: Temporomandibular Joint.

|  | **Healthy controls (n = 40)** | **Patients without Rheumatologic Pathology (n = 37)** | **p value** |
| --- | --- | --- | --- |
| Age (years) | 51.4 ± 11.6 | 58.3 ± 13.7 | 0.019 |
| Male/Female | 12/28 (80%/45.2%) | 3/34(20%/54.8%) | 0.001 |
| Subjective TMJ symptoms | 0 (0%) | 36 (100%) | <0.001 |
| Objective TMJ signs | 0 (0%) | 28 (100%) | <0.001 |
| Joint effusion | 1 (5.9%) | 16 (94.1%) | <0.001 |
| Synovial hyperplasia | 1 (16.7%) | 5 (83.3%) | 0.100 |
| Erosions | 2 (40%) | 3 (60%) | 0.667 |
| Power Doppler signal alterations | 0 (0%) | 1 (100%) | 0.481 |
| Reduction in articular cartilage thickness | 8 (40%) | 12 (60%) | 0.299 |
| Echostructural heterogeneity in articular cartilage | 0 (0%) | 1 (100%) | 0.481 |
| Cortical bone irregularities | 1 (6.7%) | 14 (93.3%) | <0.001 |
| Enthesophytes | 0 (0%) | 14 (100%) | <0.001 |
| Calcifications | 0 (0%) | 4 (100%) | 0.049 |
| Disc dislocations on the right | 0 (0%) | 4 (100%) | 0.049 |
| Disc dislocations on the left | 1 (25%) | 3 (75%) | 0.346 |

**Supplementary table S11.** Univariate comparative analysis between primary FMS *versus* HC group.

TMJ: Temporomandibular Joint.

|  | **Healthy controls (n = 40)** | **Fibromyalgia (n = 33)** | **p value** |
| --- | --- | --- | --- |
| Age (years) | 51.4 ± 11.6 | 59.7 ± 14.2 | 0.008 |
| Male/Female | 12/28 (100%/45.9%) | 0/33 (0%/54.1%) | <0.001 |
| Subjective TMJ symptoms | 0 (0%) | 33 (100%) | <0.001 |
| Objective TMJ signs | 0 (0%) | 18 (100%) | <0.001 |
| Joint effusion | 1 (8.3%) | 11 (91.7%) | 0.001 |
| Synovial hyperplasia | 1 (33.3%) | 2 (66.7%) | 0.586 |
| Erosions | 2 (66.7%) | 1 (33.3%) | 1.000 |
| Power Doppler signal alterations | 0 (0%) | 2 (100%) | 0.201 |
| Reduction in articular cartilage thickness | 8 (42.1%) | 11 (57.9%) | 0.284 |
| Echostructural heterogeneity in articular cartilage | 0 (0%) | 1 (100%) | 0.452 |
| Cortical bone irregularities | 1 (12.5%) | 7 (87.5%) | 0.019 |
| Enthesophytes | 0 (0%) | 10 (100%) | <0.001 |
| Calcifications | 0 (0%) | 3 (100%) | 0.088 |
| Disc dislocations on the right | 0 (0%) | 2 (100%) | 0.201 |
| Disc dislocations on the left | 1 (33.3%) | 2 (66.7%) | 0.586 |

**References**

1. Crincoli V, Anelli MG, Quercia E, Piancino MG, Di Comite M. Temporomandibular Disorders and Oral Features in Early Rheumatoid Arthritis Patients: An Observational Study. *Int J Med Sci* (2019) 16(2):253-63. Epub 2019/02/13. doi: 10.7150/ijms.28361.

2. Crincoli V, Cannavale M, Cazzolla AP, Dioguardi M, Piancino MG, Di Comite M. Temporomandibular Disorders and Oral Features in Idiopathic Inflammatory Myopathies (Iims) Patients: An Observational Study. *Int J Med Sci* (2021) 18(14):3158-70. Epub 20210705. doi: 10.7150/ijms.45226.

3. Crincoli V, Di Comite M, Di Bisceglie MB, Fatone L, Favia G. Temporomandibular Disorders in Psoriasis Patients with and without Psoriatic Arthritis: An Observational Study. *Int J Med Sci* (2015) 12(4):341-8. Epub 2015/05/29. doi: 10.7150/ijms.11288.

4. Maranini B, Mandrioli S, Ciancio G, Fabbian F, Galiè M, Govoni M. Temporomandibular Joint Involvement in Psoriatic Arthritis: A Prospective Clinical and Ultrasonographic Study. *Clin Exp Rheumatol* (2024) 42(1):39-47. Epub 20230803. doi: 10.55563/clinexprheumatol/pfi2ql.

5. Crincoli V, Di Comite M, Guerrieri M, Rotolo RP, Limongelli L, Tempesta A, et al. Orofacial Manifestations and Temporomandibular Disorders of Sjogren Syndrome: An Observational Study. *Int J Med Sci* (2018) 15(5):475-83. Epub 20180308. doi: 10.7150/ijms.23044.
